# A Real‐Time Mobile AI‐Assisted System for Skin Disease Prescreening: A Technical Feasibility Study

**DOI:** 10.1002/hsr2.72616

**Published:** 2026-06-07

**Authors:** Utshob Sutradhar, Priyankar Biswas, Tapos Chandra Saha, Sumon Hossain, Mahmud Iftekhar Asef

**Affiliations:** ^1^ Department of Electrical and Electronic Engineering Gopalganj Science and Technology University Gopalganj Bangladesh; ^2^ Department of Computer Science and Engineering American International University‐Bangladesh (AIUB) Dhaka Bangladesh

**Keywords:** android application, deep learning, prescreening, skin disease classification, tensorflow lite, YOLO11

## Abstract

**Background and Aims:**

Skin diseases remain a widespread health concern, particularly in areas where access to dermatological care is limited. With the growing availability of smartphones and advances in deep learning, mobile‐based image analysis offers a practical option for early‐stage screening. This study aims to develop and evaluate a lightweight, real‐time mobile AI‐assisted system for skin disease prescreening on Android devices.

**Methods:**

An Android application, SkinLearn, was developed by integrating a YOLO11‐based image classification model optimized for on‐device inference using TensorFlow Lite. The model was trained on a publicly available seven‐class skin disease dataset and further evaluated on the PAD‐UFES‐20 skin cancer dataset to examine its ability to generalize across datasets. A YOLOv8 model was also implemented under the same conditions for comparison.

**Results:**

The proposed YOLO11 model achieved a top‐1 accuracy of 99.2% on the seven‐class dataset and 71.4% on the PAD‐UFES‐20 dataset, outperforming the YOLOv8 baseline in both cases. The mobile implementation enabled real‐time, offline inference with low computational overhead, demonstrating efficient deployment in resource‐constrained environments. However, the observed performance drop across datasets highlights challenges related to domain shift, class imbalance, and variability in imaging conditions.

**Conclusion:**

These findings support the technical feasibility of lightweight deep learning models such as YOLO11 for accessible mobile skin disease prescreening. However, the observed performance drop on PAD‐UFES‐20 indicates that further validation across more diverse datasets and clinical settings is needed before any real‐world clinical use is considered.

## 1 Introduction

Skin diseases represent one of the most common health concerns worldwide, affecting individuals across age groups and geographic regions. Conditions ranging from inflammatory disorders to skin cancers may benefit from timely clinical assessment and referral [1]. However, access to dermatological expertise remains limited in many low‐resource settings. With the increasing availability of smartphones and recent advances in artificial intelligence (AI), mobile image‐based systems have emerged as a promising avenue for accessible skin disease prescreening and decision support [2].

Deep learning models, particularly convolutional neural networks and related visual recognition architectures, have shown strong performance in medical image analysis. In dermatology, several studies have reported encouraging results for lesion classification under controlled benchmark settings [3]. Among fast visual models, the You Only Look Once (YOLO) family is attractive because it enables efficient single‐pass feature extraction and prediction. However, many existing implementations still require substantial computational resources, depend on cloud inference, or are evaluated on only one dataset, which limits their suitability for mobile and resource‐constrained deployment [4, 5, 6].

Despite these advances, several practical gaps remain. First, many skin image models are not optimized for offline execution on smartphones. Second, strong single‐dataset results do not necessarily translate into stable cross‐dataset performance, especially when image quality, lighting, acquisition devices, lesion distributions, or class balance differ. Third, many published systems use language that suggests clinical diagnostic utility despite the absence of prospective validation or clinician‐in‐the‐loop evaluation [7, 8]. Accordingly, this study focuses on technical feasibility rather than clinical diagnosis.

The objectives of this study are:

- To develop a YOLO11‐based framework for real‐time skin disease image classification optimized for mobile deployment.
- To integrate the trained model into an Android application (SkinLearn) for offline, on‐device prescreening.
- To evaluate the technical performance of YOLO11 on two benchmark skin image datasets, including the PAD‐UFES‐20 dataset.
- To compare the performance of YOLO11 against a YOLOv8 baseline under the same experimental setup.
- To examine the feasibility of AI‐enabled mobile prescreening in resource‐constrained environments while explicitly acknowledging current limitations.

The remainder of the paper is organized as follows. Section Methods, 2 reviews relevant literature on mobile and deep learning‐based skin disease classification. Section Results, 3 presents the proposed methodology, including the YOLO11 architecture, training setup, and Android deployment. Section Conclusion, 4 reports the experimental results and comparative analysis. Section 5 concludes the paper and outlines limitations and future work.

## 2 Related Works

Many deep learning techniques have been studied for the classification of skin diseases. For example, a CNN architecture with nine convolutional and two fully connected layers achieved an accuracy rate of 91.07% on ten diseases [9]. A Fast R‐CNN‐based model assigned to classify skin diseases also achieved an accuracy rate of 90% at a loss value of 0.3 [10]. In a CNN hybrid system that implemented cloud and offline diagnosis, an accuracy of 83% was reported together with dermatologist interactions [11].

In terms of behaviors, mobile architectures and MobileNets were commonly used in mobile applications. For example, a TensorFlow Lite‐based Android app achieved 74.27% accuracy in classifying seven conditions [12]. In a project documenting a method for developing an Android app, the researchers used MobileNets and fine‐tuning and data augmentation methods to achieve an overall accuracy of 94.4% on dermatologic conditions [13]. A combination of an LSTM model with MobileNetV2 trained on the dataset HAM10000 achieved over 85% accuracy and demonstrated better performance efficiency [14]. A hybrid framework also achieved 98.89% accuracy on HAM10000 dataset [15]. By modifying Faster R‐CNN and integrating it with MobileNetV2, it achieved an overall accuracy of 87.2% when identifying melanoma (MEL) and actinic keratosis (ACK) for Android applications [16]. A MobileNetV2‐based Android app that employed smartphone images showed a detection accuracy of 95% and a classification accuracy of 70% [17]. A rescaled MobileNet with a partial hybrid loss function achieved an average accuracy rate of 94.76% from the images that had colors [18]. Another Android app used the Firebase ML Kit and relied on an SSD‐MobileNetV2 architecture trained on the model, which yielded an accuracy rate of 93.9% [19]. A CNN model based on federated learning maintained the privacy of users and achieved 90% accuracy in the mobile environment [20].

YOLO‐based models have produced promising results. A YOLOv11 model based on EfficientNetB0 and ResNet50 and trained using 4753 Roboflow images achieved an overall average precision of 89.8% (90% for precision and 88% precision for recall), but this did not have offline mobile deployment [21]. ViScan was built using a YOLOv10x with TensorFlow.js on the HAM10000 and achieved a mAP of 89.2%, 84.2% precision, and an F1 score of 85.2% [22]. A YOLOv8‐based assistant outperformed YOLOv3/v4 and SVM with 84% accuracy and 77.1% recall [23]. Out of five variants of YOLOv8 that were trained on HAM10000, the YOLOv8x‐cls achieved the best (~ 86.2%) accuracy and precision (~ 82.1%), while the YOLOv8n‐cls was found to have a 0.5 ms inference window, which is suitable for mobile use [24].

Several studies have also proposed hybrid or high‐performing models, that is, SkinScan demonstrated a tuned EfficientNetB7 that achieved 95% accuracy with an F1 score of 0.94 across seven skin cancers [25]. SkinVision combined CNN and Vision Transformer with real‐time explainability and achieved 81.5% sensitivity on the ISIC and Dermofit datasets [26]. DenseNet‐161, combined with a composite loss function was able to classify 40 different conditions in colored clinical images with a top‐1 accuracy of 75.07 and a top‐3 accuracy of 89.62 in classification [27].

New models have also focused on low‐resource models and screening models. A lightweight MEL detection model built using segmentation and feature selection achieved 89.09% sensitivity at ≥ 90% specificity on smartphones [28]. A binary classifier to differentiate “normal” versus “abnormal” conditions achieved 80% accuracy while targeting limited mobility use for screening purposes [29].

Overall, prior work demonstrates the promise of mobile and lightweight deep learning for dermatological image analysis, but several limitations remain: inconsistent evaluation across datasets, insufficient emphasis on offline mobile deployment, and, in some cases, overstatement of clinical applicability. These gaps motivate the present technical feasibility study.

## 3 Methodology

**Figure 1:**
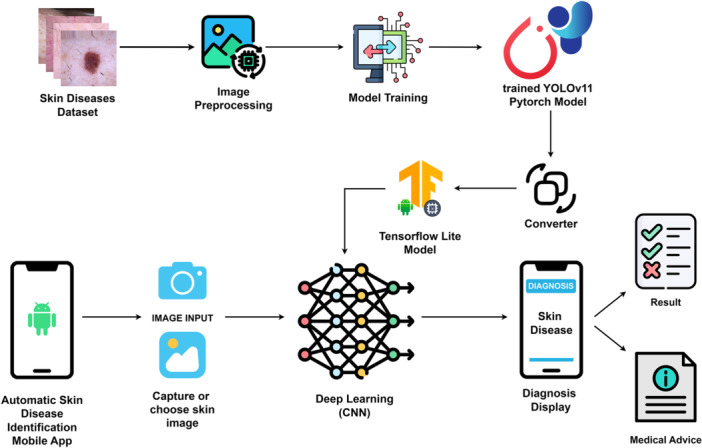
Overview of proposed system.

This study proposes a deep learning‐based framework for automated skin image classification through a smartphone application. The overall workflow includes dataset preparation, model training, conversion to TensorFlow Lite, and integration into an Android application for on‐device inference. The goal of the system is to provide rapid image‐based prescreening support rather than clinical diagnosis. Figure 1 shows the overview of the framework proposed in the proposed study.

### 3.1 Data Collection

For the first experiment, we used an open‐source skin disease image dataset [30] comprising seven classes: ACK, basal cell carcinoma (BCC), benign keratosis‐like lesions, dermatofibroma, MEL, melanocytic nevus (NEV), and vascular lesions. The dataset contains 38,459 images and is split into training, validation, and test subsets, as summarized in Table 1.

**Table 1:** Overview of skin disease dataset.

Class	Train	Validation	Test	Total
Actinic keratosis	4168	516	516	38,459
Basal cell carcinoma	4681	580	580	
Benign keratosis	4731	587	587	
Dermatofibroma	3523	436	436	
Melanoma	4731	587	587	
Melanocytic nevus	4758	590	590	
Vascular lesions	4227	524	524	
Total	30,819	3820	3820	

### 3.2 Deep Learning for Skin Diseases Classification

In this work, we employ YOLO11 as the core visual recognition model because of its computational efficiency and suitability for real‐time deployment. Although YOLO models were originally developed for object detection, their efficient backbone‐neck‐head design makes them practical for mobile image analysis tasks that require fast inference and compact deployment. The YOLO11 model is chosen because of its superior performance compared to its previous YOLO model family.

#### 3.2.1 YOLO11 Architecture

The YOLO11 architecture [31] used in this study consists of three main components: Backbone, Neck, and Head. The Backbone is responsible for hierarchical feature extraction from the input image by applying convolutional operations and feature aggregation blocks across multiple resolution levels. The Neck combines multi‐scale features through up‐sampling, concatenation, and fusion operations to preserve both fine‐grained and high‐level semantic information. The Head performs the final prediction by generating class probabilities from the fused feature maps. This backbone‐neck‐head design enables efficient end‐to‐end processing with low inference latency, which is advantageous for mobile deployment.

**Figure 2:**
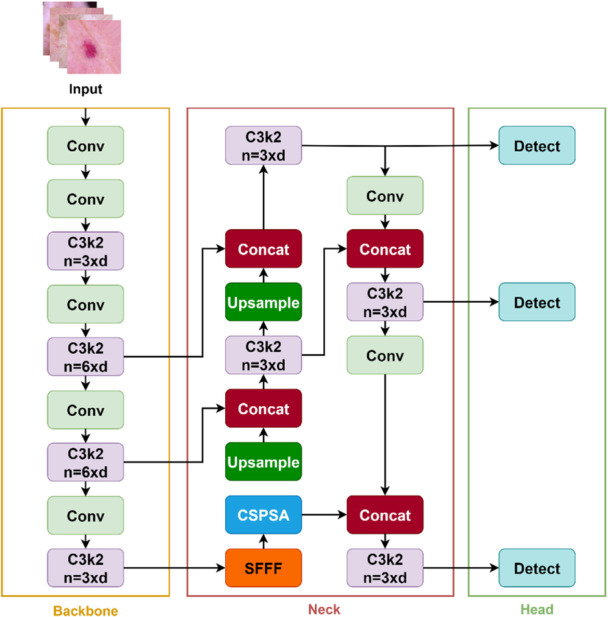
YOLO11 model architecture.

In the present implementation, an input image of size 640 × 640 × 3 is processed through convolutional layers and C3k2 blocks to extract discriminative lesion features. The Spatial Pyramid Pooling Fast (SPPF) module expands the receptive field and improves contextual representation across lesions of different apparent sizes. The C2PSA module enhances feature fusion by combining cross‐stage partial connections with attention‐guided weighting, allowing the model to emphasize more informative image regions. Together, these components improve the trade‐off between computational efficiency and classification performance, making YOLO11 suitable for lightweight mobile image analysis. The system architecture is illustrated in Figure 2.

### 3.3 Training Setup

Training was conducted in Google Colab using an NVIDIA Tesla T4 GPU (15.36 GB VRAM) with mixed‐precision computation enabled. For the seven‐class dataset, the model was trained for 14 epochs using an input size of 640 × 640 pixels and a batch size of 32. This configuration was selected based on convergence behavior observed during preliminary experiments. Training beyond 14 epochs did not provide meaningful improvement in validation performance, while longer training increased the gap between training and validation behavior, suggesting a greater risk of overfitting. Conversely, fewer epochs resulted in incomplete convergence. Therefore, 14 epochs provided a practical balance between learning capacity, generalization, and computational efficiency for the seven‐class dataset. The data split ratio was 80:10:10 for training, validation, and testing, respectively. For PAD‐UFES‐20, the model was trained for 50 epochs with the same image size and batch size to account for the smaller and more imbalanced dataset.

### 3.4 Android Application Development

**Figure 3:**
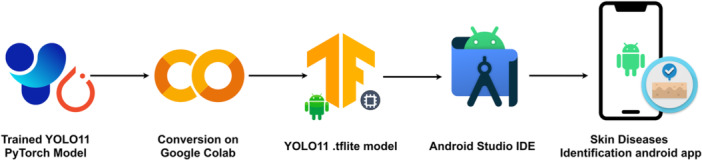
App development flow with deep learning model.

**Figure 4:**
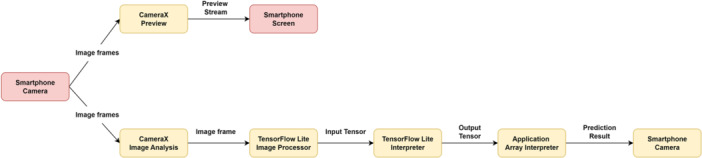
Workflow of developed YOLO11 based android app.

In a YOLO11‐based skin disease identification app, the smartphone camera streams image frames directly into the CameraX Image Analysis module, bypassing the preview if necessary for real‐time efficiency. The raw frames are preprocessed into the required format for the YOLO11 model, such as resizing to 640 × 640 and normalizing pixel values. These preprocessed frames are passed to the TensorFlow Lite Interpreter, which executes the YOLO11 model to produce an output tensor containing class IDs and confidence scores. Finally, the predicted classes are overlaid on the original frame and rendered on the smartphone screen, enabling intuitive and real‐time skin disease identification. The app development flow is shown in Figure 3. The YOLO11 model is first trained on a dataset of skin diseases using the PyTorch framework. The model is exported and converted to TensorFlow Lite format once it has achieved a suitable level of accuracy. The TensorFlow Lite Image Classification API and the tflite model are combined into an Android application using the Android Studio IDE [32]. The trained YOLO11 Pytorch model is converted to a TensorFlow Lite [33] model for mobile device deployment. Figure 4 depicts the developed app's workflow for the Android operating system (OS) [34].

## 4 Experimental Results

### 4.1 Performance on the Seven‐Class Skin Disease Dataset

**Figure 5:**
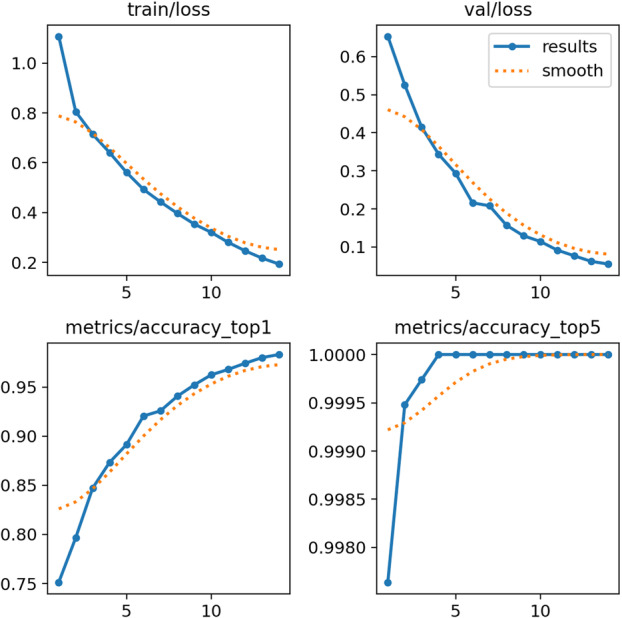
Training results of the YOLO11 model for the skin diseases dataset.

On the seven‐class dataset, the YOLO11 model demonstrated strong learning behavior, with decreasing training and validation losses over epochs and a final top‐1 accuracy of 99.2%. The high top‐5 accuracy further indicates that the relevant class was consistently ranked among the strongest predictions. These results suggest that the proposed configuration can learn discriminative features effectively within this benchmark setting. However, benchmark performance alone should not be interpreted as evidence of clinical readiness. Figure 5 shows the training results of the model.

**Figure 6:**
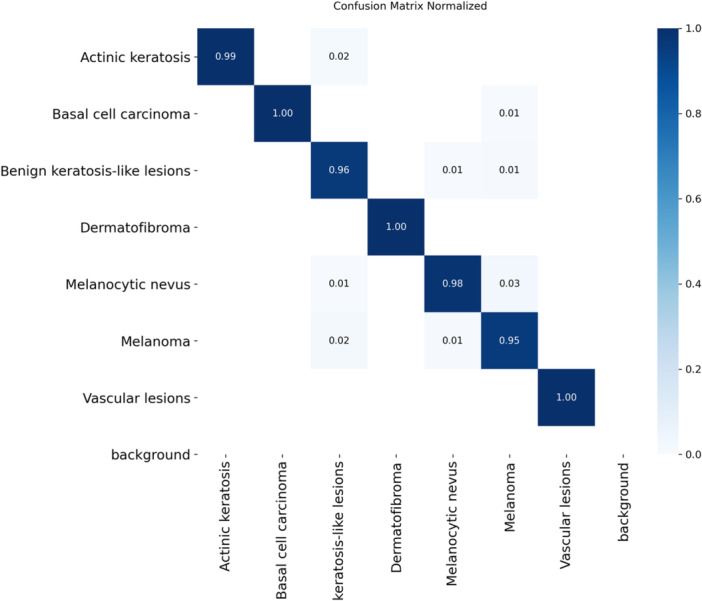
Normalized confusion matrix for skin diseases classification.

The confusion matrix illustrated in Figure 6 demonstrates the model's ability to effectively classify several key dermatological conditions, with strong prediction counts for categories such as ACK (513), BCC (579), Dermatofibroma (563), and Vascular lesions (580). This indicates robust performance in identifying these common and visually important lesion categories. While some misclassifications occur between visually similar classes, such as Melanocytic NEV and MEL, suggesting that the model captures meaningful patterns in the data. Overall, these results suggest that the model can serve as a supportive tool for image‐based prescreening and technical decision support, particularly for prevalent lesion types. However, these findings should not be interpreted as evidence of clinical diagnostic validity.

**Figure 7:**
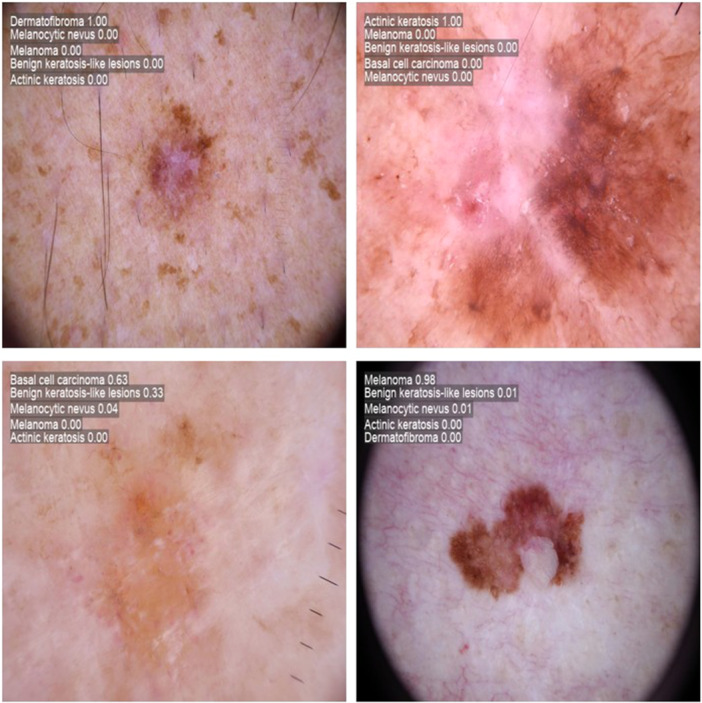
Sample classification results of the proposed YOLO11 model on the skin disease dataset.

Figure 7 presents sample classification results generated by the proposed YOLO11 model, which achieved a top‐1 accuracy of 99.2% on the general skin disease dataset. Each image displays the model's predicted probabilities across five common dermatological conditions: ACK, BCC, Benign keratosis‐like lesions, Melanocytic NEV, and MEL. The high‐confidence predictions, such as MEL (0.98) and ACK (1.00), demonstrate the model's strong capability in differentiating between visually similar lesions. These results highlight the potential of YOLO11 for accurate real‐time image‐based skin lesion prescreening on mobile platforms.

### 4.2 Comparative Analysis With YOLOv8

**Figure 8:**
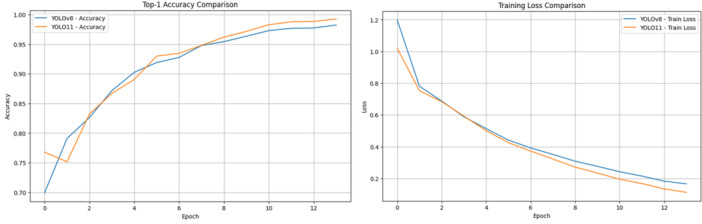
Accuracy and training loss comparison of YOLO11 with YOLOv8 model for skin disease dataset.

For the general skin disease dataset, the YOLO11 model outperformed the YOLOv8 model with an overall classification accuracy of 99.2% for all classes after training of 14 epochs. The YOLOv8 showed an accuracy of 98.2%, as shown in Figure 8. The training setups for both models were the same.

**Figure 9:**
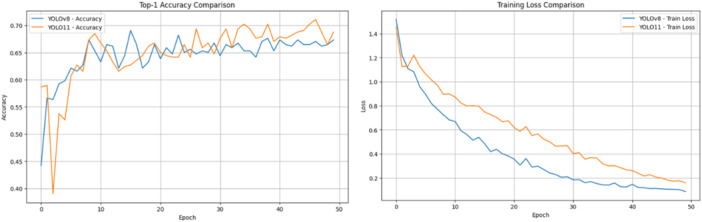
Accuracy and training loss comparison of YOLO11 with YOLOv8 model for PAD‐UFES‐20 dataset.

For the PAD‐UFES‐20 skin cancer dataset, the YOLO11 model also showed better performance with higher accuracy than the YOLOv8 model, as illustrated in Figure 9. The YOLO11 model showed an accuracy of 71.4%, while the YOLOv8 model showed 69.1%. Both models were trained for 50 epochs with the same fine‐tuning parameters with an image size of 640 × 640 and a batch size of 32. Here, the YOLOv8 model showed slightly better performance on loss validation than YOLO11.

### 4.3 Experiment Results of YOLO11 Model Classification on an Android Device

**Figure 10:**
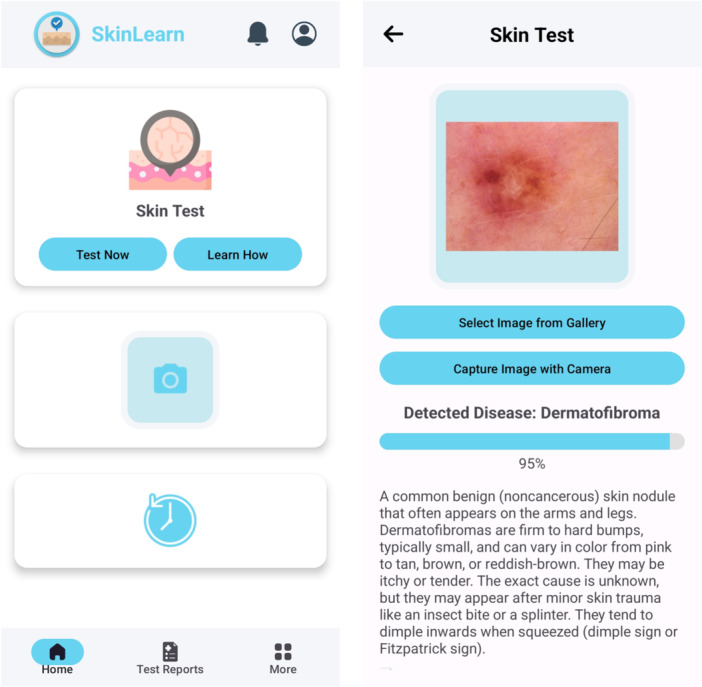
YOLO11 based android app—SkinLearn.

The SkinLearn app, depicted in Figure 10, demonstrates the technical feasibility of deploying the YOLO11 model for real‐time image‐based skin lesion prescreening on an Android device. In the example shown, the system classified Dermatofibroma with 95% confidence. The app supports both gallery uploads and real‐time camera capture, which improves usability for offline screening and educational purposes. The descriptive output is intended only to assist user understanding of the predicted class and should not be interpreted as clinical advice. Overall, the Android implementation confirms that lightweight deep learning models can be deployed efficiently for mobile prescreening, although broader validation on diverse datasets.

### 4.4 Classification Performance on the PAD‐UFES‐20 Skin Cancer Dataset

To further examine cross‐dataset performance, YOLO11 was also trained and evaluated on the PAD‐UFES‐20 skin cancer dataset [35]. The class distribution is highly imbalanced, with particularly low representation in some categories such as MEL. This imbalance should be considered when interpreting performance. The PAD‐UFES‐20 dataset includes six classes of lesions, a total of 2298 images. These are ACK, BCC, MEL, NEV, squamous cell carcinoma (SCC), and seborrheic keratosis (SEK), which are summarized in Table 2.

**Table 2:** Distribution of the PAD‐UFES‐20 dataset used in this study.

Class	Train	Validation	Total
ACK	620	110	2298
BCC	718	127	
MEL	44	8	
NEV	207	37	
SCC	163	29	
SEK	200	35	
Total	1952	346	

**Figure 11:**
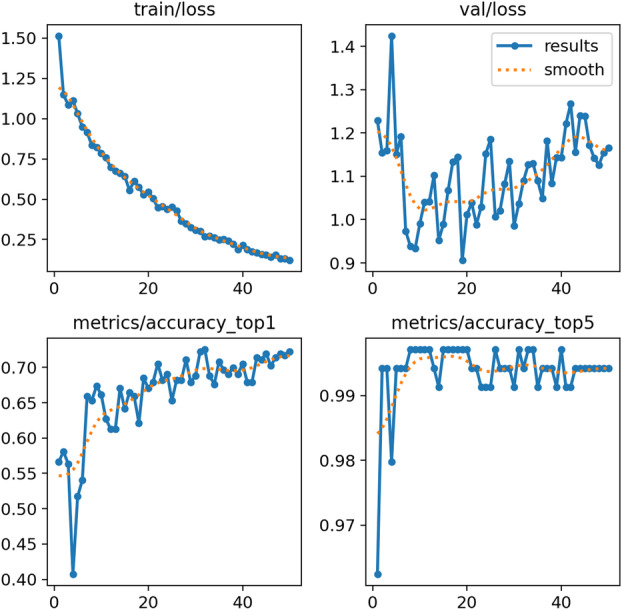
Training results of YOLO11 model for skin cancer (PAD‐UFES‐20) dataset.

The training results of the YOLO11 model on the PAD‐UFES‐20 dataset are presented in Figure 11. The dataset was divided into training and validation subsets using an 85:15 ratio across the six classes. The model achieved an accuracy of 71.4% on PAD‐UFES‐20 after 50 training epochs. Although this result remained slightly better than the YOLOv8 baseline in our comparison, the marked drop relative to the first dataset indicates weaker cross‐dataset performance. Likely contributing factors include class imbalance, differences in image acquisition conditions, lesion appearance variability, and domain shift between datasets. Therefore, the PAD‐UFES‐20 result should be interpreted as a realistic stress test of technical feasibility rather than as evidence of robust generalization across clinical environments.

As shown in Table 3, YOLO11 achieved the highest accuracy among the selected comparative methods listed here. However, because studies may differ in preprocessing, data split protocols, or auxiliary inputs, this comparison should be interpreted cautiously. Even so, the result suggests that YOLO11 is a competitive lightweight option for mobile‐oriented skin image classification.

**Table 3:** Comparative results with YOLO11 for skin disease classification task.

Study	Method	Dataset	Class	Precision [%]	Recall [%]	F1‐score [%]	Accuracy [%]
[36]	VGGNet19	PAD‐UFES‐20	6	54.72	53.11	50	55.75
	ResNet50	PAD‐UFES‐20	6	67.66	63	64.4	70.29
	InceptionV3	PAD‐UFES‐20	6	60.73	59.39	59.78	67.7
[37]	Mobilenetv2	PAD‐UFES‐20	6	—	—	—	70
[38]	Vggnet16 + SVM	PAD‐UFES‐20	6	—	—	—	53.24
	Vggnet16 + SVM	PAD‐UFES‐20	6	—	—	—	53.24
	Vggnet16 + MLP	PAD‐UFES‐20	6	—	—	—	61
	Mobilenetv2 + SVM	PAD‐UFES‐20	6	—	—	—	66
	Nasnetmobile + SVM	PAD‐UFES‐20	6	—	—	—	61.3
	Mobilenetv2 + MLP	PAD‐UFES‐20	6	—	—	—	61
Proposed	YOLO11	PAD‐UFES‐20	6	—	—	—	71.4

### 4.5 Limitations

This study has a number of limitations that should be taken into account. To begin with, the experiments were based on publicly available datasets rather than clinically collected images, which may limit how well the findings translate to real‐world settings. In addition, the PAD‐UFES‐20 dataset is notably imbalanced, and this likely influenced the model's performance on less represented classes, particularly MEL. The study also did not incorporate clinician review, additional patient metadata, subgroup analysis across demographic factors, or prospective validation with real patients. These aspects remain important to address in future work and should be considered when interpreting the results.

### 4.6 Ethical, Privacy, and Regulatory Considerations

This study relied solely on publicly available datasets and did not include patient recruitment, clinical intervention, or access to identifiable personal health data. The proposed Android application runs entirely offline on the device, which helps limit privacy concerns that can arise from cloud‐based data transfer. However, while this design supports better data privacy, it does not by itself ensure clinical safety or meet regulatory requirements. Any practical use in real‐world settings would require further validation, involvement of medical professionals, and adherence to relevant healthcare and medical device regulations. Therefore, this work should be viewed as a preliminary technical study rather than a clinically approved diagnostic solution.

## 5 Conclusion

This study introduced SkinLearn, a YOLO11‐based Android application for real‐time image‐based skin lesion prescreening on mobile devices. Across two public datasets, YOLO11 showed strong performance on the seven‐class benchmark dataset and modestly better performance than the YOLOv8 baseline on PAD‐UFES‐20. These findings support the technical feasibility of lightweight, offline, on‐device deep learning for mobile prescreening in resource‐constrained environments.

At the same time, the substantial performance drop on PAD‐UFES‐20, the class imbalance of that dataset, and the absence of clinical validation indicate that the present system should be regarded as a technical feasibility prototype rather than a diagnostic tool. Future work should evaluate the framework on more diverse and clinically representative datasets, including expert assessment and failure‐case analysis, and integrate explainability methods to improve transparency and trustworthiness before any clinical deployment is considered.

## Author Contributions

**Utshob Sutradhar:** conceptualization, methodology, software, writing – original draft, formal analysis, validation. **Priyankar Biswas:** conceptualization, methodology, visualization, writing – original draft, writing – review and editing, formal analysis, supervision, validation. **Tapos Chandra Saha:** writing – review and editing. **Sumon Hossain:** writing – review and editing. **Mahmud Iftekhar Asef:** data curation, writing – review and editing.

## Funding

The authors received no specific funding for this work.

## Conflicts of Interest

The authors declare no conflicts of interest.

## Transparency Statement

The corresponding author (Priyankar Biswas) affirms that this manuscript is an honest, accurate, and transparent account of the study being reported; that no important aspects of the study have been omitted; and that any discrepancies from the study as planned have been explained.

## Data Availability

The data that support the findings of this study are openly available in SKIN DISEASES DATASET PROCESSED at https://www.kaggle.com/datasets/ismailpromus/skin-diseases-dataset-processed. The datasets used in this study are publicly available and cited in the manuscript as [30] and [35], including the Skin Diseases Dataset Processed and PAD‐UFES‐20 datasets.
